# [^18^F]FDG Uptake and Expression of Immunohistochemical Markers Related to Glycolysis, Hypoxia, and Proliferation in Indeterminate Thyroid Nodules

**DOI:** 10.1007/s11307-022-01776-4

**Published:** 2022-10-17

**Authors:** Elizabeth J. de Koster, Adriana C. H. van Engen-van Grunsven, Johan Bussink, Cathelijne Frielink, Lioe-Fee de Geus-Oei, Benno Kusters, Hans Peters, Wim J. G. Oyen, Dennis Vriens, On behalf of the EFFECTS trial study group, Romana T. Netea-Maier, Jan W. A. Smit, Johannes H. W. de Wilt, Jan Booij, Eric Fliers, Tamira K. Klooker, Eveline W. C. M. van Dam, Koen M. A. Dreijerink, Pieter G. H. M. Raijmakers, Boen L. R. Kam, Robin P. Peeters, John F. Verzijlbergen, Maarten O. van Aken, Piet L. Jager, G. Sophie Mijnhout, Wilbert B. van den Hout, Alberto M. Pereira Arias, Johannes Morreau, Marieke Snel, Lioe-Ting Dijkhorst-Oei, John M. H. de Klerk, Bas Havekes, D. Cristina Mitea, Stefan Vöö, Catharine B. Brouwer, Pieter S. van Dam, Ferida Sivro, Erik T. te Beek, Max C. W. Jebbink, Gysele S. Bleumink, Vanessa J. R. Schelfhout, Ruth G. M. Keijsers, Iris M. M. J. Wakelkamp, Adrienne H. Brouwers, Thera P. Links, Bart de Keizer, Rachel S. van Leeuwaarde, Johannes J. Bonenkamp, A. Rogier T. Donders, Jurgen J. Fütterer

**Affiliations:** 1 Department of Medical Imaging, Nuclear Medicine, Radboud University Medical Centre, Nijmegen, the Netherlands; 2 Department of Pathology, Radboud University Medical Centre, Nijmegen, the Netherlands; 3 Department of Radiation Oncology, Radiotherapy & OncoImmunology Laboratory, Radboud University Medical Center, Nijmegen, Netherlands; 4 Department of Radiology, Section of Nuclear Medicine, Leiden University Medical Center, Leiden, the Netherlands; 5 Biomedical Photonic Imaging Group, University of Twente, Enschede, the Netherlands; 6 Department of Radiology and Nuclear Medicine, Rijnstate Hospital, Arnhem, the Netherlands; 7 Department of Biomedical Sciences and Humanitas Clinical and Research Centre, Department of Nuclear Medicine, Humanitas University, Milan, Italy

**Keywords:** Thyroid Nodule, [^18^F]FDG-PET/CT, Immunohistochemistry, Glycolysis, Glucose Metabolism

## Abstract

**Purpose:**

The current study explored the association between 2-[^18^F]fluoro-2-deoxy-D-glucose ([^18^F]FDG) uptake and the quantitative expression of immunohistochemical markers related to glucose metabolism, hypoxia, and cell proliferation in benign and malignant thyroid nodules of indeterminate cytology.

**Procedures:**

Using a case–control design, 24 patients were selected from participants of a randomized controlled multicenter trial (NCT02208544) in which [^18^F]FDG-PET/CT and thyroid surgery were performed for Bethesda III and IV nodules. Three equally sized groups of [^18^F]FDG-positive malignant, [^18^F]FDG-positive benign, and [^18^F]FDG-negative benign nodules were included. Immunohistochemical staining was performed for glucose transporters (GLUT) 1, 3, and 4; hexokinases (HK) 1 and 2; hypoxia-inducible factor-1 alpha (HIF1α; monocarboxylate transporter 4 (MCT4); carbonic anhydrase IX (CA-IX); vascular endothelial growth factor (VEGF); sodium-iodide symporter (NIS); and Ki-67. Marker expression was scored using an immunoreactive score. Unsupervised cluster analysis was performed. The immunoreactive score was correlated to the maximum and peak standardized uptake values (SUV_max_, SUV_peak_) and SUV_max_ ratio (SUV_max_ of nodule/background SUV_max_ of contralateral, normal thyroid) of the [^18^F]FDG-PET/CT using the Spearman’s rank correlation coefficient and compared between the three groups using Kruskal–Wallis tests.

**Results:**

The expression of GLUT1, GLUT3, HK2, and MCT4 was strongly positively correlated with the SUV_max_, SUV_peak_, and SUV_max_ ratio. The expression of GLUT1 (*p* = 0.009), HK2 (*p* = 0.02), MCT4 (*p* = 0.01), and VEGF (*p* = 0.007) was statistically significantly different between [^18^F]FDG-positive benign nodules, [^18^F]FDG-positive thyroid carcinomas, and [^18^F]FDG-negative benign nodules. In both [^18^F]FDG-positive benign nodules and [^18^F]FDG-positive thyroid carcinomas, the expression of GLUT1, HK2, and MCT4 was increased as compared to [^18^F]FDG-negative benign nodules. VEGF expression was higher in [^18^F]FDG-positive thyroid carcinomas as compared to [^18^F]FDG-negative and [^18^F]FDG-positive benign nodules.

**Conclusions:**

Our results suggest that [^18^F]FDG-positive benign thyroid nodules undergo changes in protein expression similar to those in thyroid carcinomas. To expand the understanding of the metabolic changes in benign and malignant thyroid nodules, further research is required, including correlation with underlying genetic alterations.

**Supplementary Information:**

The online version contains supplementary material available at 10.1007/s11307-022-01776-4.

## Introduction

Positron emission tomography/computed tomography (PET/CT) using the glucose analogue 2-[^18^F]fluoro-2-deoxy-D-glucose ([^18^F]FDG) visualizes (increased) metabolic activity in tissues and is successfully applied for the diagnosis, staging, and monitoring of many types of cancers and inflammatory disorders [1]. [^18^F]FDG-PET/CT exploits the Warburg effect, a well-known phenomenon in oncology describing the altered metabolism in malignancies: as compared to a low rate of glycolysis followed by oxidative phosphorylation in normal tissues, increased glycolysis and lactic fermentation is observed in cancer, even in the abundancy of oxygen and functioning mitochondria [2].

In differentiated thyroid carcinoma (DTC), higher [^18^F]FDG uptake is associated with more aggressive histopathology, tumor dedifferentiation, BRAF^V600E^ mutations, and other features related to an adverse prognosis [3–7].

In thyroid nodules of indeterminate cytology (defined as Bethesda classification category III or IV), a *negative* [^18^F]FDG-PET/CT accurately rules out malignancy with a 94% sensitivity and could avoid 40% of futile diagnostic surgeries for benign nodules [8, 9]. The specificity of [^18^F]FDG-PET/CT in cytologically indeterminate thyroid nodules, however, is mere 40% as many benign nodules also show increased (false positive) [^18^F]FDG uptake [9].

It is currently only partly understood which alterations in the glucose metabolism underly the differences in [^18^F]FDG uptake among various types of benign and malignant thyroid nodules.

In tumorigenesis in general, increased glucose influx into the cell by increased expression of glucose transporters (GLUT) is considered the primary mechanism behind the upregulated glucose metabolism [10–12]. Next, upregulation of the enzyme hexokinase (HK) causes increased glucose phosphorylation as the initiating step in glycolysis. As [^18^F]FDG-6-phosphate, in contrast to glucose-6-phosphate, cannot be degraded, HK activity results in increased accumulation of [^18^F]FDG [10]. Although [^18^F]FDG uptake cannot be considered a surrogate for tumor hypoxia, the expression of hypoxia-inducible factor-1 alpha (HIF1α) has been associated with [^18^F]FDG uptake [13–15]. HIF1α is a major glycolytic transcription factor, regulating the expression of many hypoxia- and glycolysis-related enzymes, including GLUT, monocarboxylate transporter 4 (MCT4), and carbonic anhydrase IX (CA-IX) [16]. Whereas MCT4 transports the lactate formed during (an)aerobic glycolysis out of the cell, CA-IX neutralizes the accompanying pH disturbances by regulating the reversible hydration of carbon dioxide [13, 17–19]. MCT4 and CA-IX are also upregulated by intracellular acidification resulting from lactate formation following aerobic glycolysis [16, 20].

As a part of tumor growth and progression, [^18^F]FDG uptake is also associated with increased cell proliferation, reflected by the expression of nuclear protein Ki-67, which, in turn, is associated with tumor aggressiveness [21–23]. Moreover, vascular endothelial growth factor (VEGF) promotes tumor cell growth and is one of the main factors involved in angiogenesis in cancer, induced by hypoxia through HIF1α [24, 25]. As glucose delivery is a function of perfusion, VEGF expression and [^18^F]FDG uptake are also associated in various cancer types [26, 27].

Finally, as [^18^F]FDG uptake is related to tumor dedifferentiation in thyroid carcinoma, an inverse relationship is observed between [^18^F]FDG and iodine uptake. While the glucose metabolism enhances, dedifferentiating thyroid carcinomas gradually lose their functional iodine uptake, reflected by the loss of the basal membranous expression of the sodium-iodide symporter (NIS) [28–30].

The association between [^18^F]FDG uptake and the expression of various metabolic markers has been investigated in papillary thyroid carcinoma (PTC) in a limited number of studies with mixed results [13, 21, 26, 31–34]. Studies in other benign and malignant thyroid nodules infrequently included [^18^F]FDG-PET/CT data [17–19, 22, 26, 34–37]. To the best of our knowledge, no studies were previously performed in cytologically indeterminate thyroid nodules.

In the current study, we explored the association between [^18^F]FDG uptake and the expression of immunohistochemical markers related to glucose transport, glucose metabolism, hypoxia, and cell proliferation in (hemi)thyroidectomy specimens of thyroid nodules with indeterminate cytology. We aimed to find correlations to explain why many benign thyroid nodules show increased [^18^F]FDG uptake and why the specificity of [^18^F]FDG-PET/CT is limited in indeterminate nodules, ultimately aiming to better understand the pathophysiology of these nodules.

## Material and Methods

### Study Design and Case Selection

The study included patients with a Bethesda III or IV thyroid nodule who underwent an [^18^F]FDG-PET/CT scan of the neck and had diagnostic thyroid surgery in the context of their participation in the *Efficacy of FDG-PET in Evaluation of Cytological indeterminate Thyroid nodules prior to Surgery (EfFECTS*) trial. This prospective, randomized controlled multicenter trial included 132 patients and was performed in 15 hospitals in the Netherlands between July 2015 and December 2019 (Clinicaltrials.gov: NCT02208544). Inclusion criteria and comprehensive study procedures of this trial were previously described [9]. For the current explorative study, including post hoc analyses of the trial data, an individually matched case–control design was chosen. We aimed to include 24 patients in three groups: eight true positives (TP), defined as patients with a visually [^18^F]FDG-positive and histopathologically malignant index nodule (i.e., differentiated (non-medullary) thyroid carcinoma), eight false positives (FP), defined as patients with a visually [^18^F]FDG-positive and histopathologically benign index nodule, and eight true negatives (TN), defined as patients with a visually [^18^F]FDG-negative and histopathologically benign index nodule. [^18^F]FDG-negative, histopathologically malignant index nodules (i.e., false negative [^18^F]FDG-PET/CT) are rare and were not selected for the current study [9]. Prior to any immunohistochemical study procedures, patients were selected for the current study from the original trial cohort by two of the researchers (EK and DV) based on the best possible match of individual patients between the three study groups while securing a balanced selection of histopathological diagnoses within groups, representative for the diagnoses found in the original trial and with a representative range in the degree of [^18^F]FDG uptake (expressed as the maximum standardized uptake value, or SUV_max_, in g/mL) per diagnosis [9]. Between groups, patients were matched based on sex, age at the time of the [^18^F]FDG-PET/CT scan, histopathological size of the index nodule, and the presence of Hürthle cells in the histopathology sample. TP and FP cases were additionally matched based on the SUV_max_ of the index nodule. Patients were not eligible for inclusion in the current study if the thyroid nodule was smaller than 10 mm (due to possible limitations regarding the spatial resolution of the PET/CT scanner), if there was either an [^18^F]FDG-positive hotspot within an [^18^F]FDG-positive nodule or central photopenia (to limit the possibility of sampling error), if the [^18^F]FDG-PET/CT images showed signs of thyroiditis throughout the thyroid including the index nodule, if the last fine needle aspiration cytology procedure (FNAC) and [^18^F]FDG-PET/CT scan were less than 4 weeks apart (i.e., to limit the possibility of [^18^F]FDG-positivity due to reactive tissue damage repair changes following FNAC), if no histopathology was available (i.e., following patient treatment allocation in the *EfFECTS* trial), and/or if review of the histopathology showed signs of lymphocytic thyroiditis or necrosis in the index nodule (i.e., to limit the possibility of false positive or false negative [^18^F]FDG-PET/CT readings, respectively).

The trial, including the current secondary analysis, was approved by the Medical Research Ethics Committee on Research Involving Human Subjects region Arnhem-Nijmegen, Nijmegen, the Netherlands. Written informed consent was obtained from each of the participants prior to any study activities.

### FDG-PET/CT Acquisition, Reconstruction, and Analysis

During the *EfFECTS* trial, all participants underwent a single [^18^F]FDG-PET/CT of the neck. Scans were acquired by 20 different scanners at 12 EARL-accredited study sites using a standard acquisition and reconstruction protocol in accordance with European Association of Nuclear Medicine (EANM) guidelines [1]. Patients fasted for at least 6 h, and serum glucose levels were between 4 and 11 mmol/L. PET acquisition was scheduled 60 (55–75) min after intravenous bolus administration of [^18^F]FDG. The administered activity was dependent on body weight, scan speed, bed overlap, and scanner sensitivity, equivalent to 3.45 MBq/kg (4 min/bed, < 25% bed overlap). Low-dose, non-contrast-enhanced CT (ldCT) scans were acquired for attenuation correction of PET images.

**Fig. 1:**
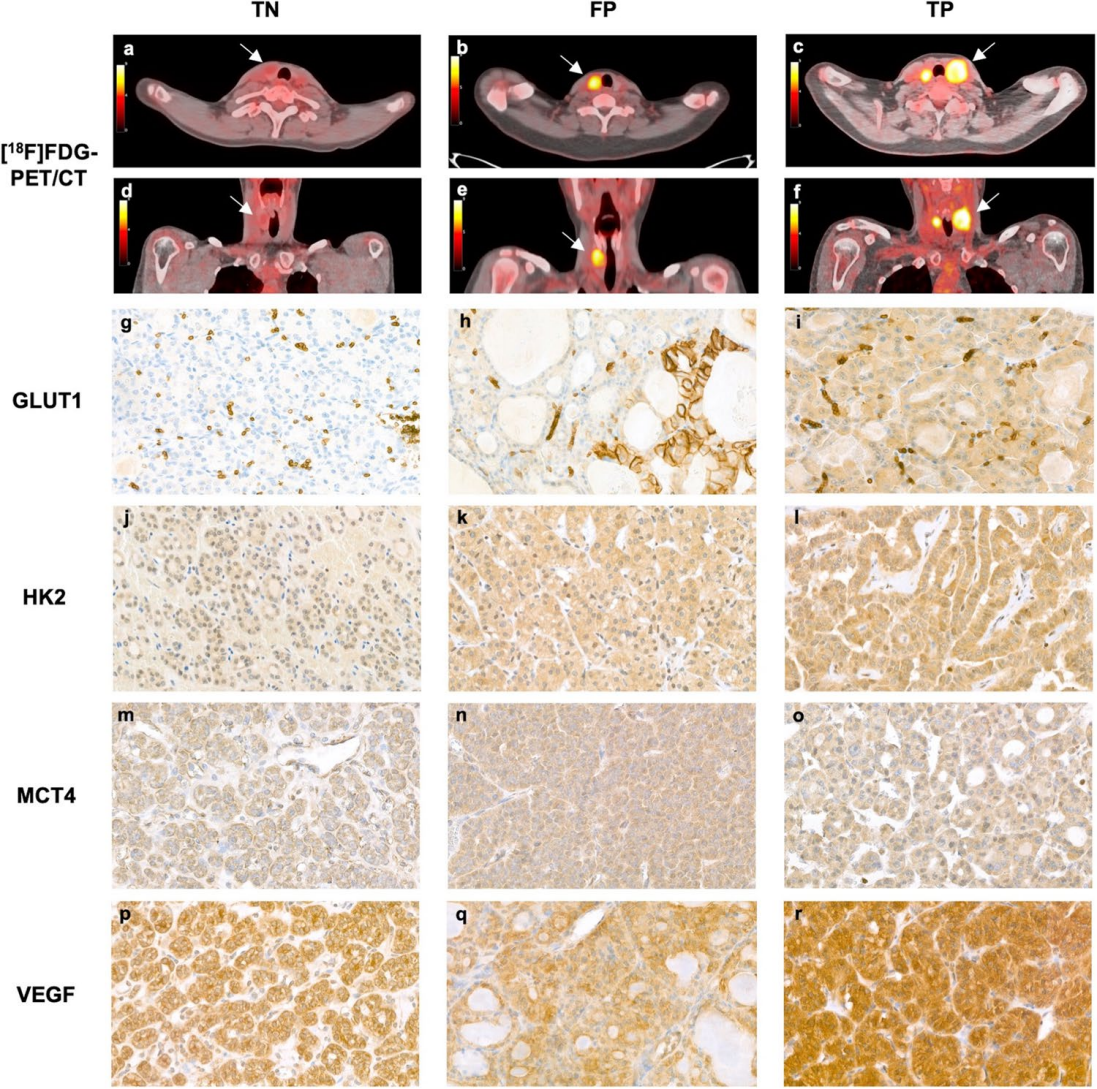
Representative [^18^F]FDG-PET/CT images and immunohistochemical staining patterns for the three groups. Transverse and coronal [^18^F]FDG-PET/CT images of a right-sided, 19-mm, visually [^18^F]FDG-negative follicular adenoma (TN group) with a SUV_max_ of 2.2 g/mL (**a**, **d**), a right-sided, 25-mm, visually [^18^F]FDG-positive follicular adenoma (FP group) with a SUV_max_ of 8.0 g/mL (**b**, **e**), and a left-sided, 40-mm, visually [^18^F]FDG-positive minimally invasive FTC (pT2N0Mx, TP group) with a SUV_max_ of 10.0 g/mL (with contralateral [^18^F]FDG-positive multinodular goiter) (**c**, **f**). Illustrative, representative microscopy images (× 40) of the immunohistochemistry stains observed in each of the groups show absent GLUT1 (**g**), weak HK2 (**j**), weak MCT4 (**m**), and intermediate VEGF (**p**) expression in TN nodules; weak cytoplasmic GLUT1 (**h**), intermediate HK2 (**k**), intermediate MCT4 (**n**), and intermediate VEGF (**q**) expression in FP nodules; and intermediate GLUT1 (**i**), intermediate HK2 (**l**), intermediate MCT4 (**o**), and strong VEGF (**r**) expression in TP nodules. In addition, several FP and TP nodules showed strong membranous GLUT1 expression in < 10% of cells (**h**). Note: the [^18^F]FDG-PET/CT images and microscopy images in each column represent multiple patients from each group to represent the average findings per group; single patients with immunohistochemistry results consistent with the group average were not available. FP, false positives. FTC, follicular thyroid carcinoma. GLUT, glucose transporter. HK, hexokinase. MCT4, Monocarboxylate transporter 4. SUV_max_, maximum standardized uptake value. TN, true negatives. TP, true positives. VEGF, vascular endothelial growth factor.

All scans were centrally assessed by two independent, experienced nuclear medicine physicians (DV, LF). They were blinded to patient allocation and all clinical and cytological data except for the ultrasonographic size and location of the index nodule, to ensure its correct identification. For the visual assessment, any focal [^18^F]FDG uptake within the thyroid that was visually higher than the physiological background [^18^F]FDG uptake of the surrounding normal thyroid tissue and that corresponded to the index nodule in size and location was considered positive (Fig. 1). Quantitative image analyses were performed using OsiriX Lite DICOM-viewer (Pixmeo SARL, Bernex, Switzerland). SUV computation was validated after each mandatory software version update. The SUV_max_ and peak SUV (SUV_peak_, defined as the maximum average SUV within a 1 cm^3^ spherical volume) of the index nodule were semi-automatically measured. Body weight-corrected values were used. The SUV_max_ ratio was calculated by dividing the SUV_max_ of the nodule by the background SUV_max_ of normal thyroid tissue in the contralateral lobe. [^18^F]FDG-positive foci in the thyroid that did not correspond to the index nodule in size and location (i.e., thyroid incidentalomas) were not analyzed in the current study.

### Histopathology

During the *EfFECTS* trial, all postoperative patient management was based on the *local* histopathological diagnosis. For scientific purposes and to limit the effect of any interobserver variability, all (hemi)thyroidectomy specimens were *centrally* reviewed by a dedicated thyroid pathologist (AE) in accordance with the WHO classification (4th edition) [38]. In case of discordance with the local diagnosis, an additional dedicated thyroid pathologist (BK) was consulted to reach consensus. Local and central histopathologists were blinded to the [^18^F]FDG-PET/CT result; the two central pathologists were also blinded to the local histopathological diagnosis. Incidentally detected (micro)carcinomas located outside the index nodule were not considered for the main outcome measure.

### Immunohistochemistry

Indirect chromogenic immunohistochemical staining was performed on 5-μm thick paraffin-embedded tissue sections on coated slide glasses. We used primary antibodies against GLUT1 (RB-9052-P, Thermo Fisher Scientific, Waltham, MA, USA; dilution 1:500), GLUT3 (RB-9096-P, Immunologic, WellMed BV, Duiven, the Netherlands; 1:300), GLUT4 (ab654, Abcam, Cambridge, UK: 1:1000), HK1 (MA5-15,680, Invitrogen™, Thermo Fisher Scientific; 1:7500), HK2 (MA5-15,679, Invitrogen™, Thermo Fisher Scientific; 1:200), HIF1α (ab2185, Abcam; 1:250), MCT4 (anti SLC16A4, HPA046986, Sigma-Aldrich®, Saint Louis, MO, USA; 1:200), CA-IX (NB100-417, Novus Biologicals™, Bio-techne, Centennial, CO, USA; 1:250), VEGF (555,036, Pharmingen™, Becton Dickinson Biosciences, San Diego, CA, USA; 1:100), NIS (ABC1453, Merck Millipore, Burlington, MA, USA; 1:2000), and Ki-67 (M724001, DAKO Agilent, Santa Clara CA, USA; 1:25).

Tissue sections were deparaffinized and rehydrated. Next, GLUT1, GLUT3, HIF1α, CA-IX, and Ki-67 staining was performed using a semi-automated immunostainer (Thermo Scientific, Labvision™ 488) following a standardized protocol according to manufacturer instructions. GLUT4, HK1, HK2, MCT4, VEGF, and NIS staining was performed manually. Detailed immunohistochemistry procedures are provided in the Supplementary data. In summary, antigen retrieval was performed, non-specific immunoreactivity was blocked, and slides were subsequently incubated with primary and secondary antibodies. Slides were incubated using 3,3-diaminobenzidine (DAB) as chromogen. Negative control samples were processed without primary antibodies. Positive control tissues were used as recommended by the manufacturer. Finally, slides were counterstained with hematoxylin and dehydrated.

All slides were reviewed to quantify the expression of the immunohistochemical markers by evaluating the DAB staining by two members of the research team (EK and AE), who were blinded to the clinical data and [^18^F]FDG-PET/CT results (Fig. 1). The staining was assessed using the Remmele and Stegner immunoreactive score (IRS) by multiplying the staining intensity (score 0–3: negative, weak, intermediate, strong) with the proportion of stain-positive cells in the index nodule (score 0–4: 0%, 1–10%, 11–50%, 51–80%, > 80%) (Supplementary Table 1) [39]. Consequently, the minimum IRS of 0 represents absent staining, and the maximum IRS of 12 represents strong staining in a majority of cells in the lesion. For the Ki-67 stain, the proliferation index was assessed as the percentage of cells with positive nuclear staining (score 1–3: < 3%, 3–5%, > 5%) among at least four representative fields of 100 tumor cells.

### Statistical Analysis

Baseline characteristics were compared between the allocated groups using Pearson’s chi-square for categorical data and one-way ANOVA or Kruskal–Wallis tests for continuous data, where appropriate. Unsupervised cluster analysis with ordered leaves was performed using the IRS of all stains and Ki-67 proliferation index and visualized in a dendrogram with heat map. The IRS of all stains was correlated with each other, the SUV_max_, SUV_peak_, and SUV_max_ ratio using the Spearman’s rank correlation coefficient with its degrees of freedom (r_s_[df]). Next, the IRS of all immunomarkers was compared between TN, FP, and TP groups using Kruskal–Wallis (omnibus) tests and visualized using violin plots. In case the Kruskal–Wallis test indicated statistical significance, Dunn’s post hoc tests were used for each pair of groups, and *p* values are presented. Counteracting for multiple comparisons was performed using Bonferroni correction; statistical significance after correction is indicated by an asterisk (*). A *p* value of 0.05 or less was considered statistically significant. Statistical analysis was performed using IBM SPSS Statistics version 27 (IBM Corp., Armonk, NY, USA) and Orange: Data Mining Toolbox version 3.30.2 (Bioinformatics Lab, University of Ljubljana, Slovenia) [40].

## Results

Twenty-four patients were included, of which 16 (67%) were female. The mean age was 53.1 ± 14.7 years. The median nodule size was 30 mm (interquartile range 24–45). SUV_max_, SUV_peak_, and SUV_max_ ratio were statistically significantly different between the groups (Table 1). These differences were significant between TN and FP and between TN and TP groups, but not between FP and TP groups.

**Table 1:** Baseline characteristics^a^

	TN	FP	TP	
	*n* = 8	*n* = 8	*n* = 8	*p*
Female	6 (75%)	5 (63%)	5 (63%)	0.83^b^
Age (years) (mean ± SD)	51.2 ± 12.3	54.0 ± 11.8	54.2 ± 20.3	0.91^c^
Nodule size on histopathology, mm (median, IQR)	33 (25–45)	26 (21–43)	35 (21–44)	0.83^d^
Thyroid function				
TSH, mU/L (median, IQR)	1.20 (0.46–2.05)	1.65 (0.98–2.80)	1.55 (1.18–1.75)	0.21^d^
fT4, pmol/L (median, IQR)	16.1 (13.6–17.4)	14.1 (13.3–15.5)	13.8 (12.0–19.8)	0.53^d^
[^18^F]FDG-PET/CT scan				
SUV_max_ (g/mL) (median, IQR)	2.1 (1.2–2.3)	7.0 (4.5–31.0)	11.1 (4.2–20.8)	** < 0.001**^**d,e**^
SUV_peak_ (g/mL) (median, IQR)	1.7 (1.0–2.1)	4.8 (4.0–24.6)	9.6 (2.8–15.8)	**0.002**^**d,e**^
SUV_max_-ratio (median, IQR)	1.0 (0.8–1.2)	4.3 (2.3–14.3)	6.2 (1.6–14.0)	** < 0.001**^**d,e**^
Histopathological diagnosis				
Hyperplastic nodule	4 (50%)	2 (25%)		
Follicular adenoma	4 (50%)	4 (50%)		
Hürthle cell adenoma		2 (25%)		
PTC			2 (25%)	
FVPTC			1 (12.5%)	
FTC, minimally invasive			3 (37.5%)	
HCC, minimally invasive			2 (25%)	
TNM stage				
T1a			0 (0%)	
T1b			2 (25%)	
T2			4 (50%)	
T3			2 (25%)	
N0/x			6 (75%)	
N1a			2 (25%)	

*FP*, false positives. *FTC*, follicular thyroid carcinoma. *FVPTC*, follicular variant PTC. *HCC*, Hürthle cell carcinoma. *IQR*, interquartile range. *PTC*, papillary thyroid carcinoma. *SD*, standard deviation. *TN*, true negatives. *TP*, true positives

^a^Individual patient characteristics are presented in Supplementary Table 2

^b^Pearson’s chi-squared

^c^One-way ANOVA

^d^Kruskal-Wallis test

^e^Post hoc analysis: SUV_max_ *p* = 0.001* between TN and FP groups, *p* < 0.001* between TN and TP groups, and *p* = 0.89 between FP and TP groups; SUV_peak_ *p* = 0.003* between TN and FP groups, *p* = 0.001* between TN and TP groups, and *p* = 0.83 between FP and TP groups; SUV_max_-ratio *p* = 0.001* between TN and FP groups, *p* < 0.001* between TN and TP groups, and *p* = 0.89 between FP and TP groups

**Fig. 2:**
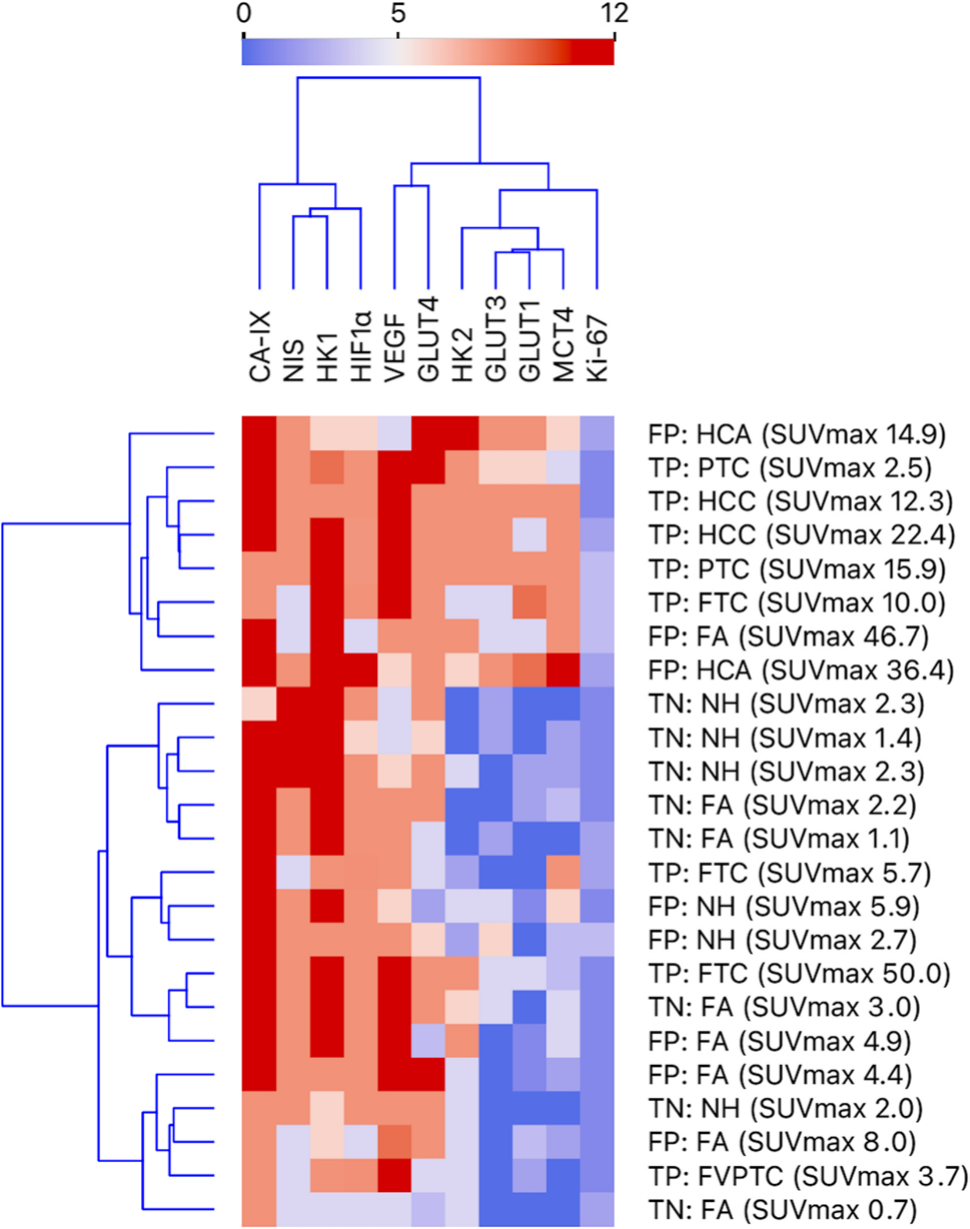
Dendrogram heatmap showing the unsupervised cluster analysis of all 11 immunohistochemical stains of the 24 thyroid nodules, including the group, histopathological diagnosis, and SUV_max_ of these nodules. The IRS is presented on a scale from 0 (dark blue, absent stain) to 12 (dark red, strong stain in < 80% of tumor cells). CA-IX, carbonic anhydrase IX. FA, follicular adenoma. FP, false positives. FTC, follicular thyroid carcinoma. FVPTC, follicular variant PTC. GLUT, glucose transporter. HCA, Hürthle cell adenoma. HCC, Hürthle cell carcinoma. HIF1α, Hypoxia-inducible factor-1 alpha. HK, hexokinase. IRS, immunoreactive score. MCT4, Monocarboxylate transporter 4. NH, nodular hyperplasia. NIS, sodium-iodide symporter. PTC, papillary thyroid carcinoma. SUV_max_, maximum standardized uptake value. TN, true negatives. TP, true positives. VEGF, vascular endothelial growth factor.

Unsupervised cluster analysis (Fig. 2) indicated fair clustering of nodules in TN, FP, and TP groups based on the IRS of the 11 immunomarkers. Many lesions with a high SUV_max_ appeared to have a high expression of multiple markers, including FP as well as TP nodules.

**Fig. 3:**
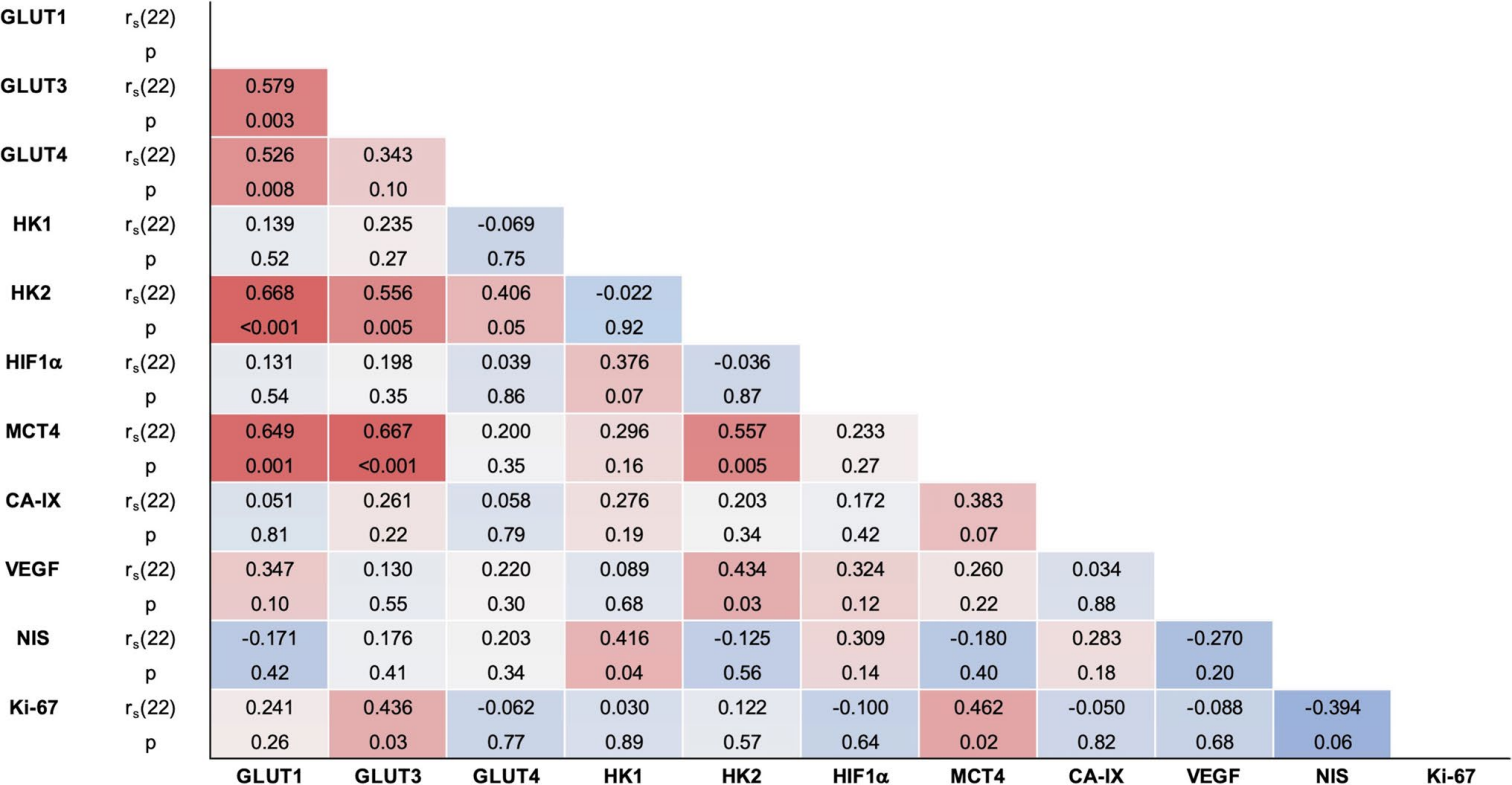
Spearman’s rank correlation coefficients (r_s_, df = 22) of the IRS of the 11 immunohistochemical markers, presented on a color scale from − 1 (dark blue) to 1 (dark red). CA-IX, carbonic anhydrase IX. GLUT, glucose transporter. HIF1a, Hypoxia-inducible factor-1 alpha. HK, hexokinase. IRS, immunoreactive score. MCT4, Monocarboxylate transporter 4. NIS, sodium-iodide symporter. VEGF, vascular endothelial growth factor.

The expression of multiple markers moderately positively correlated with each other: GLUT1 to GLUT3, GLUT4, HK2, and MCT4; GLUT3 to HK2, MCT4, and Ki-67; HK1 to NIS; HK2 to GLUT4, MCT4, and VEGF; and MCT4 to Ki-67 (Fig. 3).

The expression of GLUT1, GLUT3, HK2, and MCT4 was strongly positively correlated with the SUV_max_, SUV_peak_, and SUV_max_ ratio (Table 2). For GLUT1, both its cytoplasmic (r_s_(22) = 0.715, *p* < 0.001) and membranous (r_s_(22) = 0.450, *p* = 0.03) expressions were related to the SUV_max_. For GLUT3, only its cytoplasmic expression was correlated with the SUV_max_ (r_s_(22) = 0.649, *p* < 0.001).

**Table 2:** Correlations between the IRS and SUV

	SUV_max_		SUV_peak_		SUV_max_ ratio	
IHC stain	*r*_s_	*p*	*r*_s_	*p*	*r*_s_	*p*
GLUT1	0.731	** < 0.001**	0.731	** < 0.001**	0.720	** < 0.001**
GLUT3	0.579	**0.003**	0.556	**0.005**	0.582	**0.003**
GLUT4	0.301	0.15	0.315	0.13	0.294	0.16
HK1	0.182	0.40	0.237	0.27	0.096	0.66
HK2	0.695	** < 0.001**	0.681	** < 0.001**	0.707	** < 0.001**
HIF1α	0.102	0.64	0.111	0.61	0.124	0.56
MCT4	0.760	** < 0.001**	0.765	** < 0.001**	0.740	** < 0.001**
CA-IX	0.152	0.48	0.113	0.60	0.166	0.44
VEGF	0.373	0.07	0.393	0.054	0.359	0.09
NIS	− 0.270	0.20	− 0.270	0.20	− 0.262	0.22
Ki-67	0.319	0.13	0.275	0.19	0.354	0.09

*CA-IX*, carbonic anhydrase IX. *GLUT*, glucose transporter. *HIF1α*, Hypoxia-inducible factor-1 alpha. *HK*, hexokinase. *IRS*, immunoreactive score. *MCT4*, Monocarboxylate transporter 4. *NIS*, sodium-iodide symporter. *r*_s_ Spearman’s rank correlation coefficient (*df* = 22). *SUV*, standardized uptake value. *VEGF*, vascular endothelial growth factor

**Fig. 4:**
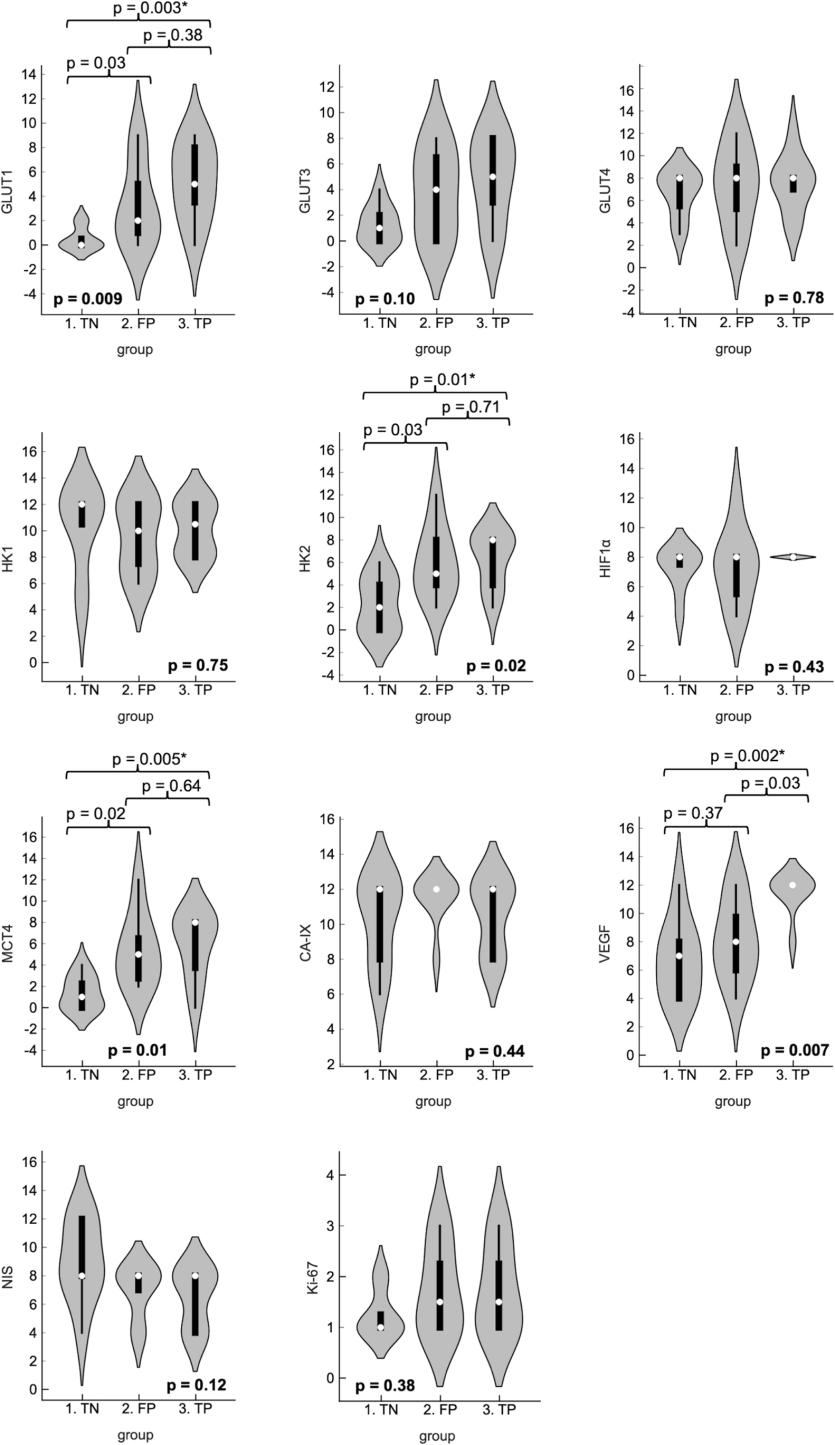
Violin plots demonstrating the between-group comparison of the IRS of all IHC stains, including the median (white dot), interquartile range (thick black whisker), range (thin black whisker), and density based on counts (kernel). The boldfaced *p* value (bottom of each plot) represents the overall *p* value (Kruskal–Wallis test). The horizontal braces and corresponding *p* values represent the *p* values between two groups (post hoc Dunn’s test). Single asterisk (*) indicates statistical significance after Bonferroni correction for multiple comparisons. CA-IX, carbonic anhydrase IX. FP, false positives. GLUT, glucose transporter. HIF1a, Hypoxia-inducible factor-1 alpha. HK, hexokinase. IRS, immunoreactive score. MCT4, Monocarboxylate transporter 4. NIS, sodium-iodide symporter. TN, true negatives. TP, true positives. VEGF, vascular endothelial growth factor.

The expression of GLUT1, HK2, MCT4, and VEGF was statistically significantly different between the TN, FP, and TP groups (Fig. 4). Post hoc analysis demonstrated that the expression of GLUT1, HK2, and MCT4 was similarly increased in FP and TP nodules as compared to the expression in TN nodules. VEGF expression was higher in TP as compared to both TN and FP nodules. VEGF expression was similar in TN and FP groups.

## Discussion

When performing and interpreting [^18^F]FDG-PET/CT scans, we should aim to understand the underlying pathophysiological processes at a cellular and molecular level. In indeterminate thyroid nodules, the specificity of [^18^F]FDG-PET/CT is limited, as many benign nodules also show increased [^18^F]FDG uptake [9]. The current explorative study demonstrated that [^18^F]FDG uptake in indeterminate thyroid nodules was positively correlated with the expression of GLUT1, GLUT3, HK2, and MCT4. [^18^F]FDG-positive benign thyroid nodules and [^18^F]FDG-positive thyroid carcinomas with indeterminate cytology showed increased expression of GLUT1, HK2, and MCT4 as compared to the expression in [^18^F]FDG-negative benign nodules. The expression of VEGF was similar in [^18^F]FDG-positive and [^18^F]FDG-negative benign nodules, which was lower than in [^18^F]FDG-positive thyroid carcinomas. GLUT3 expression was strongly correlated with the SUV metrices, but not statistically significantly different between the TN, FP, and TP groups in the current dataset. These results indicate that glucose metabolism-related alterations in the protein expression of [^18^F]FDG-positive benign thyroid nodules may be similar to alterations occurring in thyroid carcinomas, at least in those deriving from cytologically indeterminate nodules.

To the best of knowledge, our study is the first to assess immunohistochemical staining for metabolic markers in comparison to [^18^F]FDG-PET/CT results in cytologically indeterminate thyroid nodules.

The previous, heterogeneous studies of these markers in thyroid carcinomas showed mixed results, possibly hindered by their oftentimes limited sample size and statistical power [21, 26, 33, 34]. An increased expression of GLUTs and HKs was observed in PTC and to a lesser extent in follicular thyroid carcinoma (FTC) as compared to follicular adenoma (FA) and normal thyroid tissue [18, 35, 36, 41]. An association between [^18^F]FDG uptake and the increased expression of GLUTs and HKs was previously observed in DTC but remained unconfirmed in the latest studies [13, 21, 26, 31–34]. [^18^F]FDG-positive PTC showed a higher expression of HIF1α as compared to [^18^F]FDG-negative PTC [13]. Higher expression of MCT4 and CA-IX was previously found in poorly differentiated and anaplastic thyroid carcinoma as compared to PTC and FTC and in FTC as compared to FA [18, 19]. MCT4 was not previously associated to [^18^F]FDG uptake in thyroid neoplasms. Increased expression of VEGF was observed in both PTC and FTC as compared to FA and multinodular goiter [24, 33, 42]. In PTC and FTC, CA-IX, VEGF, and NIS expression appeared unrelated to [^18^F]FDG uptake [26, 33, 34]. The Ki-67 proliferation index was higher in FTC than FA [37]. In a single study, the Ki-67 proliferation index was also associated with the SUV_max_ in PTC [21].

The current study does not resolve the origin of the enhanced glucose metabolism and increased [^18^F]FDG uptake in the malignant and part of the benign thyroid nodules. In thyroid carcinomas, [^18^F]FDG uptake likely reflects metabolic alterations caused by oncogenic mutations in various pathways. In PTC, the presence of a BRAF^V600E^ mutation is associated with [^18^F]FDG uptake and with overexpression of GLUT1, GLUT3, HK2, HIF1α, MCT4, and CA-IX and loss of NIS expression [18, 32, 43, 44]. In vitro studies also suggested the influence of RAS mutations on [^18^F]FDG uptake [45]. GLUT1 expression is also regulated by the PI3K/AKT/mTOR pathway, and in FTC, loss of function of PTEN increased the membrane expression of GLUT1 in vitro [46, 47]. Thus, at least a part of the benign nodules may carry genetic alterations that cause metabolic changes similar to those in thyroid carcinomas.

Hürthle cell neoplasms are considered a separate entity among the follicular cell-derived thyroid neoplasms. They are distinct in their clinical and biological behavior and carry different genetic alterations, primarily characterized by copy number alterations [48–50]. Malignant as well as benign Hürthle cell neoplasms are almost without exception strongly [^18^F]FDG-positive, likely due to the abundance in mitochondria which is a typical feature of oncocytic cells [9, 51]. In two previous studies, the expression of GLUT1, HK2, MCT4, and CA-IX and the Ki-67 proliferation index were higher in Hürthle cell thyroid carcinoma and adenoma as compared to their non-oncocytic counterparts [19, 37]. In the current study, we included two Hürthle cell adenomas and two Hürthle cell carcinomas, all with high SUV_max_ (range 12.3 to 36.4 g/mL). Similar to literature, all four Hürthle cell lesions also showed remarkable and relatively strong expression of GLUTs, HK2, MCT4, and most other markers (Fig. 2).

In previous studies in thyroid cancer, correlations were established between various metabolic markers and signs of adverse prognosis. For example, GLUT1 overexpression was associated with tumor dedifferentiation, lymph node metastasis, and shorter overall survival [18, 22, 35, 36]. HIF1α overexpression was associated with metastasis in FTC [17]. A high Ki-67 index was correlated with aggressive malignant behavior in both PTC and FTC [23, 37]. Loss of NIS expression was associated with tumor dedifferentiation [30, 43]. In Hürthle cell carcinoma, HK2 expression was associated with a tumor size larger than 4 cm, MCT4 with extrathyroidal tumor extension, and CA-IX with vascular invasion [19]. In the current study, only a limited number of thyroid carcinoma were included, and such prognostic correlations could not be assessed.

The main limitations of the current study were its case–control design, which is susceptible to selection bias, and its limited sample size. The latter limited the statistical power and may have caused underreporting of any effects. As the study was designed as a secondary and explorative analysis of data acquired during a randomized controlled trial, the current study was not powered in advance to distinguish differences in immunohistochemical staining. In addition, during the interpretation of the data, it was assumed that the degree of immunohistochemical expression of metabolic markers positively correlated with the functional activity of these proteins. This assumption may not necessarily be correct and may be considered a general limitation of immunohistochemical studies. For example, immunohistochemical expression of NIS is not an accurate predictor of radioiodine uptake [29].

In conclusion, the positive correlations between [^18^F]FDG uptake and GLUT1, GLUT3, HK2, and MCT4 expression and differential expression of GLUT1, HK2, and MCT4 in [^18^F]FDG-positive benign thyroid nodules and [^18^F]FDG-positive thyroid carcinomas as compared to [^18^F]FDG-negative benign nodules in the current study suggest that these [^18^F]FDG-positive benign nodules undergo metabolic changes similar to those in thyroid carcinomas. Further studies in larger populations are required to confirm the findings of the current explorative study and unravel the underlying cellular mechanisms. A more extensive assessment, including a comparison of the genetic alterations, protein expression, and [^18^F]FDG-PET/CT results, could aid to connect genotype to phenotype. A separate analysis is recommended for Hürthle cell and non-Hürthle cell nodules.

## Supplementary Information

Below is the link to the electronic supplementary material.

Supplementary file1 (DOCX 35 KB)
