# Supplementary material for: [18F]FDG Uptake and Expression of Immunohistochemical Markers Related to Glycolysis, Hypoxia, and Proliferation in Indeterminate Thyroid Nodules
Source: Mol Imaging Biol. 2022 Oct 17;25(3):483–94. doi: 10.1007/s11307-022-01776-4 (PMC10172288; doi:10.1007/s11307-022-01776-4)
Supplement: Supplementary file 1 — Supplementary file1 (DOCX 35 KB) [file 11307_2022_1776_MOESM1_ESM.docx]

**Supplementary data**

**FDG uptake and expression of immunohistochemical markers related to glycolysis and hypoxia in indeterminate thyroid nodules**

Elizabeth J. de Koster, Adriana C.H. van Engen-van Grunsven, Johan Bussink, Cathelijne Frielink, Lioe-Fee de Geus-Oei, Benno Kusters, Hans Peters, Wim J.G. Oyen, Dennis Vriens on behalf of the *EfFECTS trial* study group.

**Table of contents**

**EfFECTS trial study group 3.**

Trial steering committee 3.

Local principal investigators 3.

Study safety committee 5.

**Supplementary Material and Methods**

HK1, HK2, GLUT4, VEGF, NIS and MCT4 staining 6.

Supplementary table 1. Scoring of stain positivity for 8.

the individual immunohistochemical marker

**Supplementary Results**

Supplementary table 2. Characteristics of the 24 included patients in the three groups 9.

**EfFECTS trial study group**

**Trail steering committee**

- Prof. dr. L.F. de Geus-Oei, MD PhD (**project leader**), *Leiden University Medical Center, Department of Radiology, Section of Nuclear Medicine, Leiden, the Netherlands; Radboud University Medical Centre, Department of Radiology and Nuclear Medicine, Nijmegen, the Netherlands*
- Prof. dr. W.J.G. Oyen, MD PhD (**principal investigator**), *Radboud University Medical Centre, Department of Radiology and Nuclear Medicine, Nijmegen, the Netherlands; Rijnstate Hospital, Department of Radiology and Nuclear Medicine, Arnhem, the Netherlands; Department of Biomedical Sciences and Humanitas Clinical and Research Centre, Department of Nuclear Medicine, Humanitas University, Milan, Italy*
- Dr. D. Vriens, MD PhD (**principal investigator**), *Leiden University Medical Center, Department of Radiology, Section of Nuclear Medicine, Leiden, the Netherland.*
- E.J. de Koster, MD (**junior investigator**), *Radboud University Medical Centre, Department of Radiology and Nuclear Medicine, Nijmegen, the Netherlands*

**Local principal investigators**

**Radboud university medical centre, Nijmegen, the Netherlands**

- Dr. A.C.H. van Engen-van Grunsven, MD PhD, *Department of Pathology*
- E.J. de Koster, MD, *Department of Radiology and Nuclear Medicine*
- Dr. B. Küsters, MD PhD, *Department of Pathology*
- Prof. dr. R.T. Netea-Maier, MD PhD, *Department of Internal Medicine, Division of Endocrinology*
- Prof. dr. J.W.A. Smit, MD PhD, *Department of Internal Medicine, Division of Endocrinology*
- Prof. dr. J.H.W. de Wilt, MD PhD, *Department of Surgical Oncology*

**Amsterdam University Medical Centers, Amsterdam, the Netherlands**

*Location Academic Medical Center*

- Prof. dr. J. Booij, MD PhD, *Department of Radiology and Nuclear Medicine*
- Prof. dr. E. Fliers, MD PhD, *Department of Endocrinology and Metabolism*
- Dr. T.K. Klooker, MD PhD, *Department of Endocrinology and Metabolism*

*Location VU University Medical Center*

- Dr. E.W.C.M. van Dam, MD PhD, *Department of Internal Medicine, Division of Endocrinology*
- Dr. K.M.A. Dreijerink, MD PhD, *Department of Internal Medicine, Division of Endocrinology*
- Dr. P.G.H.M. Raijmakers, MD PhD, *Department of Radiology and Nuclear Medicine*

**Erasmus University Medical Centre, Rotterdam, the Netherlands**

- Dr. B.L.R. Kam, MD PhD, *Department of Nuclear Medicine*
- Prof. dr. R.P. Peeters, MD PhD, *Department of Internal Medicine*
- Prof. dr. J.F. Verzijlbergen, MD PhD, *Department of Nuclear Medicine*

**Haga Hospital, The Hague, the Netherlands**

- Dr. M.O. van Aken, MD PhD, *Department of Internal Medicine*

**Isala Hospital, Zwolle, the Netherlands**

- Prof. dr. P.L. Jager, MD PhD, *Department of Nuclear Medicine*
- Dr. G.S. Mijnhout, MD PhD, *Department of Internal Medicine*

**Leiden University Medical Center, Leiden, the Netherlands**

- Prof. dr. L.F. de Geus-Oei, MD PhD, *Department of Radiology, Section of Nuclear Medicine*
- Dr. W.B. van den Hout, PhD, *Department of Biomedical Data Sciences-Medical Decision Making*
- Prof. dr. A.M. Pereira Arias, MD PhD, *Department of Internal Medicine, Division of Endocrinology*
- Prof. dr. J. Morreau, MD PhD, *Department of Pathology*
- Dr. M. Snel, MD PhD, *Department of Internal Medicine, Division of Endocrinology*
- Dr. D. Vriens, MD PhD, *Department of Radiology, Section of Nuclear Medicine*

**Meander Medical Centre, Amersfoort, the Netherlands**

- Dr. L.T. Dijkhorst-Oei, MD PhD, *Department of Internal Medicine*
- Dr. J.M.H. de Klerk, MD PhD, *Department of Nuclear Medicine*

**Maastricht University Medical Centre, Maastricht, the Netherlands**

- Dr. B. Havekes, MD PhD, *Department of Internal Medicine, Division of Endocrinology*
- Dr. D.C. Mitea, MD PhD, *Department of Radiology and Nuclear Medicine*
- Dr. S. Vöö, MD PhD, *Department of Radiology and Nuclear Medicine*

**OLVG Hospital, Amsterdam, the Netherlands**

- Dr. C.B. Brouwer, MD PhD, *Department of Internal Medicine*
- Dr. P.S. van Dam, MD PhD, *Department of Internal Medicine*
- Dr. F. Sivro, MD PhD, *Department of Nuclear Medicine*

**Reinier de Graaf Hospital, Delft, the Netherlands**

- Dr. E.T. te Beek, MD PhD, *Department of Nuclear Medicine*
- Dr. M.C.W. Jebbink, MD PhD, *Department of Internal Medicine*

**Rijnstate Hospital, Arnhem, the Netherlands**

- Dr. G.S. Bleumink, MD PhD, *Department of Internal Medicine*
- Prof. dr. W.J.G. Oyen, MD PhD, Department of Radiology and Nuclear Medicine
- Dr. V.J.R. Schelfhout, MD PhD, Department of Radiology and Nuclear Medicine

**St. Antonius Hospital, Nieuwegein, the Netherlands**

- Dr. R.G.M. Keijsers, MD PhD, *Department of Nuclear Medicine*
- Dr. I.M.M.J. Wakelkamp, MD PhD, *Department of Internal Medicine*

**University Medical Centre Groningen, Groningen, the Netherlands**

- Dr. A.H. Brouwers, MD PhD, *Department of Nuclear Medicine and Molecular Imaging*
- Prof. dr. T.P. Links, MD PhD, *Division of Endocrinology, Department of Internal Medicine*

**University Medical Centre Utrecht, Utrecht, the Netherlands**

- Dr. B. de Keizer, MD PhD, *Department of Radiology and Nuclear Medicine*
- Dr. R.S. van Leeuwaarde, MD PhD, *Department of Endocrine Oncology*

**Study safety committee**

- Dr. J.J. Bonenkamp, MD PhD, *Department of Surgical Oncology, Radboud University Medical Centre, Nijmegen, The Netherlands.*
- Dr. A.R.T. Donders, PhD, *Department for Health Evidence, Radboud University Medical Centre, Nijmegen, the Netherlands*
- Prof. dr. J.J. Fütterer, Phd, *Department of Radiology and Nuclear Medicine, Radboud University Medical Centre, Nijmegen, The Netherlands.*

**Supplementary Material and Methods**

***HK1, HK2, GLUT4, VEGF, NIS and MCT4 staining***

Tumor tissue slides were deparaffinized, rehydrated and washed with ethanol series (100%, 100%, 70%, and 50%) and demi water. Antigen retrieval was performed with 10 mM citrate pH 6.0 in a PT module (Thermo Fisher Scientific, Waltham, MA, USA) (10 minutes at 96°C) for HK1, HK2, GLUT4, VEGF and NIS. Antigen retrieval for the MCT4 staining was performed during 30 minutes at 98°C. After cooling, the slides were washed twice with demi water and 3 times with 10 mM phosphate buffered saline (PBS).

Next, for HK1, HK2, GLUT4, VEGF and NIS, endogenous peroxidase activity was quenched with 3% hydrogen peroxide (H_2_O_2_) in PBS in the dark for 10 minutes. Again, slides were washed twice with demi water and 3 times with 10 mM PBS. For GLUT4, slides were washed twice with demi water, twice with 10 mM PBS and once with PBS including 0.05% Tween-20. Non-specific interactions were blocked using incubation in normal goat serum (20% in PBS) for 30 minutes for HK1, HK2, GLUT4, VEGF, and NIS, and 5% for 45 minutes for MCT4. Primary antibody diluted in PBS/1% bovine serum albumin (BSA) was applied for 60 minutes for HK1, HK2, GLUT4, and VEGF and overnight at 4°C for NIS and MCT4. Next, the slides were washed 3 times with 10 mM PBS. For GLUT4, slides were washed twice with 10 mM PBS and once with PBS including 0.05% Tween-20.

Next, for MCT4, endogenous peroxidase activity was quenched with 3% hydrogen peroxide (H_2_O_2_) in PBS in the dark for 10 minutes. Slides were washed twice with demi water and 3 times with 10 mM PBS.

Secondary antibody incubation was performed using Bright Vision poly HRP-GAM/Rb IgG (VWR KDPVO110 HRP, Immunologic, WellMed BV, Duiven, the Netherlands) for 30 minutes for HK1, HK2, GLUT4, and NIS and 60 minutes for MCT4. For VEGF, Goat-anti-mouse IgG2b/PO was used (1090-05, Southern Biotech, Birmingham, AL, USA, diluted 1:200). Next, the slides were washed 3 times with 10 mM PBS. For GLUT4, slides were washed twice with 10 mM PBS and once with PBS including 0.05% Tween-20. Finally, the slides were incubated with 3,3-diaminobenzidine (DAB) (BS04-500, Immunologic, VWR, Dublin, Ireland) for 8 minutes for HK1, HK2, GLUT4, VEGF and NIS, and 25 minutes for MCT4. After washing three times with tap water, the slides were counterstained with hematoxylin for 5 seconds, and washed with tap water for 10 minutes and twice with demi water. Then, slides were washed and dehydrated in ethanol series (50%, 70%, 100%) followed by xylene, mounted with a coverslip (Permount^TM^, Fisher Scientific, Waltham. MA, USA), and dried overnight in the fume hood.

Negative control samples were processed without the primary antibody. Positive control tissues were used according to the protocols of the manufacturers.

***GLUT1, GLUT3, HIF1α, CA-IX, and Ki-67***

Tumor tissue slides were deparaffinized, rehydrated and washed three times with 99% ethanol and then with demi water. Antigen retrieval was performed using a PT module (Thermo Scientific, Waltham, MA, USA) boiling the sections for 10 minutes at 95°C using a retrieval buffer solution (high pH, DAKO Agilent, Santa Clara, CA, USA). Next, slides for GLUT1, GLUT3, HIF1α, CA-IX, and Ki-67 staining were loaded in a semi-automated immunostainer (Labvision^TM^ 488, Thermo Scientific, Waltham, MA, USA) and stained following a standard protocol according to the instructions of the manufacturer. In brief, sections were washed with wash buffer (EnVision^TM^ wash buffer, DAKO Agilent, Santa Clara, CA, USA), incubated with primary antibody for 60 minutes, washed with wash buffer, incubated for 30 minutes with horseradish peroxidase (HRP, EnVision^TM^, DAKO Agilent, Santa Clara, CA, USA), washed with wash buffer, incubated for 10 minutes with DAB (EnVision^TM^, DAKO Agilent, Santa Clara, CA, USA), and washed with water. Sections were then removed from the immunostainer. Next, they were counterstained using hematoxylin and dehydrated (Prisma®, Sakura Finetek Europe, Alphen aan den Rijn, the Netherlands).

**Supplementary table 1**

Scoring of stain positivity for the individual immunohistochemical marker

| **Immunohistochemical marker** | **Considered positive stain** |
| --- | --- |
| GLUT1 | Cytoplasmic and/or membranous stain |
| GLUT3 | Cytoplasmic and/or membranous stain |
| GLUT4 | Cytoplasmic and/or membranous stain |
| HK1 | Cytoplasmic and/or membranous stain |
| HK2 | Cytoplasmic and/or membranous stain |
| HIF1α | Cytoplasmic and/or nuclear (homogeneously and darkly stained nuclei) stain |
| MCT4 | Cytoplasmic and/or membranous stain |
| CA-IX | Cytoplasmic and/or membranous stain |
| VEGF | Cytoplasmic and/or membranous stain |
| NIS | Cytoplasmic and/or membranous stain |
| Ki-67 | nuclear (homogeneously and darkly stained nuclei) stain |

CA-IX, carbonic anhydrase IX. GLUT, glucose transporter. HIF1α, Hypoxia-inducible factor-1 alpha. HK, hexokinase. MCT4, Monocarboxylate transporter 4. NIS, sodium-iodide symporter. VEGF, vascular endothelial growth factor.

**Supplementary table 2**

Characteristics of the 24 included patients in the three groups

| **Patient no.** | **Group** | **Sex** | **Age** | **Cytology result** | **Visually [^18^F]FDG-avid** | **SUV_max_ (g/mL)** | **Size (mm)** | **Histopathological diagnosis** |
| --- | --- | --- | --- | --- | --- | --- | --- | --- |
| 1 | TN | M | 61 | AUS/FLUS | No | 0.7 | 25 | Follicular adenoma |
| 2 | TN | M | 72 | AUS/FLUS | No | 3.0 | 33 | Follicular adenoma |
| 3 | TN | F | 55 | FN/SFN | No | 2.2 | 19 | Follicular adenoma |
| 4 | TN | F | 39 | AUS/FLUS | No | 1.1 | 60 | Follicular adenoma |
| 5 | TN | F | 37 | AUS/FLUS | No | 2.3 | 29 | Hyperplastic nodule |
| 6 | TN | F | 50 | AUS/FLUS | No | 1.4 | 30 | Hyperplastic nodule |
| 7 | TN | F | 40 | AUS/FLUS | No | 2.0 | 45 | Hyperplastic nodule |
| 8 | TN | F | 53 | FN/SFN | No | 2.3 | 38 | Hyperplastic nodule |
| 9 | FP | M | 63 | HCN/SHCN | Yes | 36.4 | 35 | Hürthle cell adenoma |
| 10 | FP | M | 49 | HCN/SHCN | Yes | 14.9 | 20 | Hürthle cell adenoma |
| 11 | FP | F | 52 | HCN/SHCN | Yes | 46.7 | 45 | Follicular adenoma, dd Hürthle cell adenoma |
| 12 | FP | F | 53 | AUS/FLUS | Yes | 8.0 | 25 | Follicular adenoma, dd hyperplastic nodule |
| 13 | FP | F | 66 | FN/SFN | Yes | 4.4 | 25 | Follicular adenoma |
| 14 | FP | M | 52 | FN/SFN | Yes | 4.9 | 45 | Follicular adenoma, dd hyperplastic nodule |
| 15 | FP | F | 66 | AUS/FLUS | Yes | 5.9 | 27 | Hyperplastic nodule |
| 16 | FP | F | 30 | HCN/SHCN | Yes | 2.7 | 20 | Hyperplastic nodule |
| 17 | TP | M | 76 | HCN/SHCN | Yes | 22.4 | 45 | HCC |
| 18 | TP | F | 82 | HCN/SHCN | Yes | 12.3 | 45 | HCC |
| 19 | TP | F | 30 | FN/SFN | Yes | 50.0 | 20 | FTC, minimally invasive |
| 20 | TP | F | 56 | FN/SFN | Yes | 10.0 | 40 | FTC, minimally invasive |
| 21 | TP | F | 61 | AUS/FLUS | Yes | 5.7 | 30 | FTC, minimally invasive |
| 22 | TP | M | 23 | FN/SFN | Yes | 15.9 | 24 | PTC |
| 23 | TP | M | 50 | AUS/FLUS | Yes | 2.5 | 39 | PTC |
| 24 | TP | F | 53 | FN/SFN | Yes | 3.7 | 13 | FVPTC |

AUS/FLUS, atypia of undetermined significance or follicular lesion of undetermined significance. dd, differential diagnosis. F, female. FN/SFN, (suspicious for a) follicular neoplasm. FP, false-positives. FTC, follicular thyroid carcinoma. FVPTC, follicular variant PTC. HCC, Hürthle cell carcinoma. HCN/SHCN, (suspicious for a) Hürthle cell neoplasm. M, male. PTC, papillary thyroid carcinoma. TN, true-negatives. TP, true-positives
